# A Novel Characteristic Gastric Mucus Named “Web-like Mucus” Potentially Induced by Vonoprazan

**DOI:** 10.3390/jcm13144070

**Published:** 2024-07-11

**Authors:** Hiroaki Kaneko, Hiroki Sato, Yuichi Suzuki, Aya Ikeda, Hirofumi Kuwashima, Ryosuke Ikeda, Takeshi Sato, Kuniyasu Irie, Soichiro Sue, Shin Maeda

**Affiliations:** 1 Department of Gastroenterology, Yokohama City University Graduate School of Medicine, 3-9 Fukuura Kanazawa-Ku, Yokohama 236-0004, Japan; hero5722@yokohama-cu.ac.jp (H.K.); sato.hir.wa@yokohama-cu.ac.jp (H.S.); ys291238@gmail.com (Y.S.); ikedaa@yokohama-cu.ac.jp (A.I.); ryosuke@yokohama-cu.ac.jp (R.I.); tak_sato@yokohama-cu.ac.jp (T.S.); k_irie@yokohama-cu.ac.jp (K.I.); ssue@yokohama-cu.ac.jp (S.S.); 2 Yokohama Hodogaya Central Hospital, 43-1 Kamadai-Chou Hodogaya-Ku, Yokohama 240-8585, Japan; hirofumi_kuwashima_2118@yahoo.co.jp

**Keywords:** acid secretion, gastric mucus adhesion, PPI, vonoprazan, web-like mucus

## Abstract

**Background:** In the absence of Helicobacter pylori (HP) infection, a characteristic gastric mucus adhesion may appear during the use of vonoprazan. We named this novel characteristic mucus “web-like mucus” (WLM). This study aimed to determine the incidence and risk factors for WLM. **Methods:** Between January 2017 and January 2022, 5665 patients were enrolled in this study. The patients were divided into a proton-pump inhibitor (PPI)-prescribed group (*n* = 2000), a vonoprazan-prescribed group (*n* = 268), and a no-PPI/vonoprazan-prescribed (*n* = 3397) group, and the presence of WLM was examined. After excluding four patients with autoimmune gastritis, the remaining 264 patients in the vonoprazan group were divided into WLM and non-WLM groups, and their clinical features were analyzed. **Results:** A total of 55 (21%) patients had WLM, all in the vonoprazan-prescribed group. There were no significant differences in factors such as, sex, age, chronic kidney disease, diabetes mellitus, HP eradication history, smoking, or alcohol consumption between the WLM and non-WLM groups. The median duration from the start of vonoprazan administration to the endoscopic detection of WLM was 2 (1–24) months. **Conclusions:** WLM appears to be a characteristic feature in patients treated with vonoprazan.

## 1. Introduction

Vonoprazan (Takecab^®^; Takeda Pharmaceutical Co., Ltd., Tokyo, Japan) is a potassium-competitive acid blocker (PCAB) that was developed and approved for use in Japan in February 2015 [1]. Vonoprazan belongs to a class of drugs that reversibly inhibit gastric acid output through K^+^-competitive ionic binding to H^+^/K^+^-ATPase [2]. Vonoprazan has been reported to be effective for treating reflux esophagitis [3], eradication of *Helicobacter pylori* (HP) [4], treating gastric and duodenal ulcers [3], and even treating post-endoscopic submucosal dissection ulcers [3]. Furthermore, vonoprazan is effective in preventing peptic ulcer recurrence during low-dose aspirin therapy and in patients receiving long-term nonsteroidal anti-inflammatory drugs (NSAIDs) [5,6,7]. Therefore, the number of vonoprazan prescriptions has steadily increased in recent years.

However, recent studies have reported vonoprazan-associated changes in gastric mucosa, such as cracked and cobblestone-like gastric mucosa, white globe appearance lesions in the noncancerous stomach, stardust gastric mucosa, and gastric mucosal redness [8,9,10,11]. Similarly, we have noticed a characteristic mucus-like sticky substance in patients receiving vonoprazan in our clinical daily endoscopy examinations. Therefore, in this study, we aimed to define this mucus and investigate its characteristics in detail.

## 2. Methods

### 2.1. Definitions of Web-like Mucus

**Figure 1:**
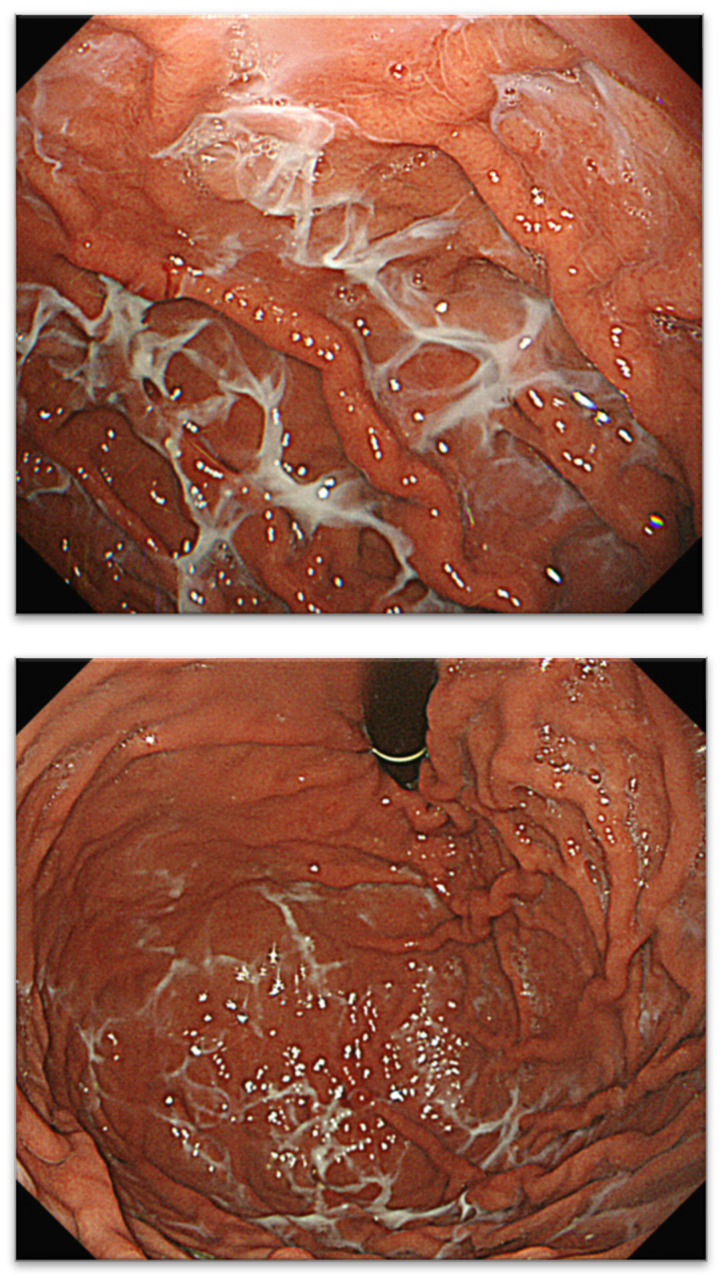
Typical case with WLM and spiderweb. WLM, web-like mucus.

**Figure 2:**
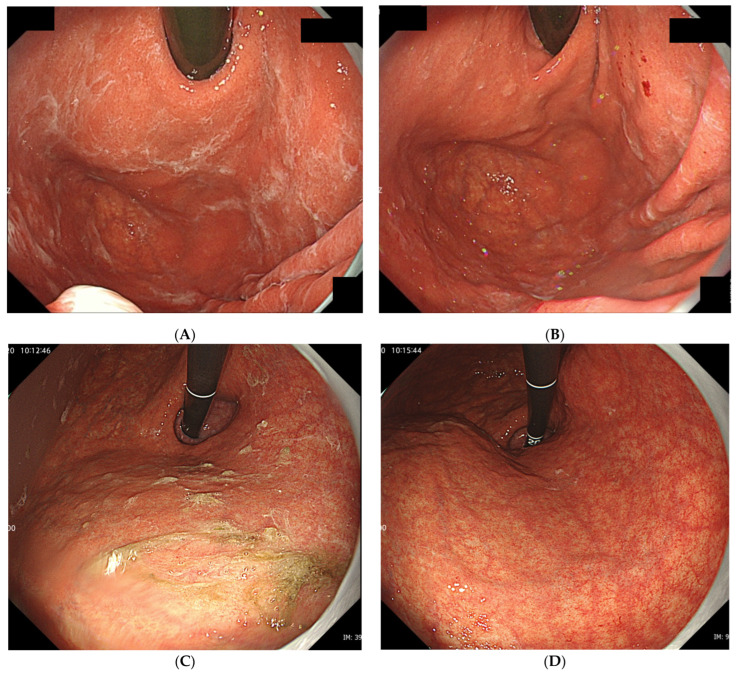
(**A**) Typical gastric mucus due to *H. pylori* infection that (**B**) can be easily removed by washing or brushing endoscopically. (**C**) Typical sticky and adherent, dense mucus seen in autoimmune gastritis. (**D**) Although it adheres strongly to the mucosa, it can be removed by careful washing.

Since 2016, we have detected several patients with characteristic gastric mucus adhesion on endoscopy that resembled a spider’s web. Thus, we named this characteristic mucus “web-like mucus” (WLM). We defined WLM as follows: endoscopically white and transparent mucus with a spider web-like appearance, which is difficult to remove endoscopically by washing thoroughly with a syringe or water pump for the endoscope (OFP-2; Olympus Optical Co., Ltd., Yokohama, Japan) (Figure 1). In particular, removal difficulty is essential to distinguish it from mucus adhesion due to HP infection (Figure 2A,B). Furthermore, WLM differs from the sticky, adherent, dense mucus seen in autoimmune gastritis [12] in terms of color tone and removal difficulty (Figure 2C,D).

### 2.2. Patients

**Figure 3:**
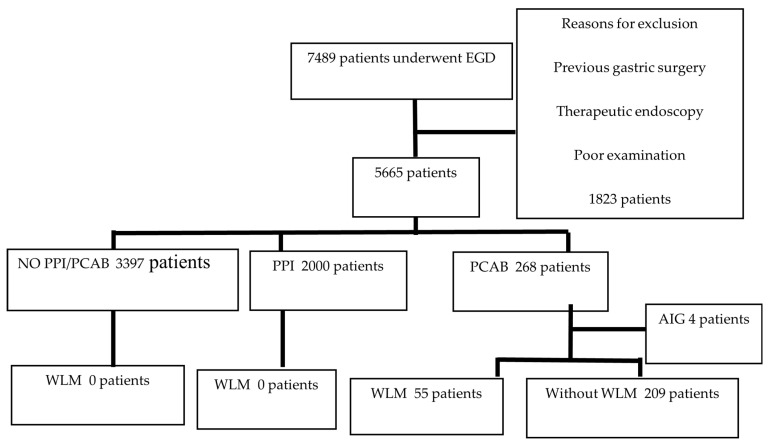
Flow diagram of patient selection. AIG, autoimmune gastritis; EGD, esophagogastroduodenoscopy; PPI, proton-pump inhibitor; PCAB, potassium competitive acid blocker; WLM, web-like mucus.

This was a single-center retrospective study. Between January 2017 and January 2022, 7489 patients underwent upper gastrointestinal endoscopy at the Yokohama City University Graduate School of Medicine in Yokohama, Japan. Patients who underwent therapeutic endoscopy, emergency endoscopy, or previous gastric surgery and those who could not be retrospectively evaluated in detail were excluded; 5665 patients were finally enrolled. The patients were classified into proton-pump inhibitor (PPI)-prescribed (*n* = 2000), vonoprazan-prescribed (*n* = 268), and no-PPI/vonoprazan-prescribed (*n* = 3397) groups, and the presence of WLM was retrospectively compared. In addition, after excluding four cases of autoimmune gastritis from the vonoprazan-prescribed group, the remaining 264 cases were divided into WLM and non-WLM groups (Figure 3), and the following factors were compared: sex, age, diabetes mellitus, chronic kidney disease, HP eradication history, duration of vonoprazan therapy, smoking, alcohol consumption, and medication history, such as steroids, rebamipide, anticoagulants, and NSAIDs.

### 2.3. Endoscopic Procedure and Diagnosis

Although there were no specific dietary restrictions on the day before the endoscopy, all patients fasted from the morning of the day of examination. Essentially, the patients were asked to continue all prescribed medications except diabetes medications; however, the final decision on which medications were to be continued was left to the physician who ordered the examination. We used 3 mL of 2% dimethicone (Barytgen antifoaming solution; Fushimi Pharmaceutical Co., Ltd., Kagawa, Japan) and 0.5 g of pronase (Pronase MS; Kaken Pharmaceutical Co., Ltd., Tokyo, Japan) immediately before endoscopy to help defoam and remove extra mucus in the upper gastrointestinal tract. All patients received local pharyngeal anesthesia using 8–40 mg of an 8% lidocaine pump spray (Xylocaine; Sand Pharma, Co., Ltd., Tokyo, Japan) unless there were drug allergies. During endoscopy, the patients who wished to be sedated were placed under conscious sedation with midazolam (Sand Pharma, Co., Ltd.). Endoscopy was performed using GIF-Q260J, GIF-H260Z, GIF-H290, or GIF-H290Z (Olympus Optical Co., Ltd., Yokohama, Japan). According to the Kimura–Takemoto classification, we identified atrophic gastritis as none for C0, closed type for C1 and C3, and open type for O1 and O3 [13]. We also subdivided the stomach into upper, middle, and lower thirds. All endoscopic images were recorded using an endoscopic filling system (NEXUS; Fujifilm Medical, Tokyo, Japan). We retrospectively examined the location of the WLM, active HP infection, cobblestone-like gastric mucosa, stardust gastric mucosa, gastric mucosal redness, multiple white and flat elevated lesions, fundic gland polyps, hyperplastic polyps, and atrophic gastritis using the endoscopic images and reports. The images were reviewed by two endoscopists (HK and RI) blinded to patient information.

The risks and benefits of routine endoscopy were explained to all the patients, and written informed consent was obtained individually. The ethics committee of the Yokohama City University Graduate School of Medicine approved the study protocol.

### 2.4. Statistical Analysis

The baseline characteristics of the patients are expressed as means ± standard deviations, and categorical data are expressed as proportions. Non-normally distributed continuous variables are expressed as medians and interquartile ranges and were compared using the Wilcoxon rank-sum test. Using the Mann–Whitney U test, Pearson’s chi-squared test, the Kruskal–Wallis test, or Fisher’s exact test, we assessed the differences between groups as appropriate. The logistic regression model was used to calculate the adjusted odds ratios (ORs) with 95% confidence intervals (CIs) for risks of WLM. All *p*-values were two-sided, and *p* < 0.05 was considered statistically significant. All statistical data were analyzed using SPSS Statistics 28.0.1 (IBM).

## 3. Results

### 3.1. Patients Characteristics

**Figure 4:**
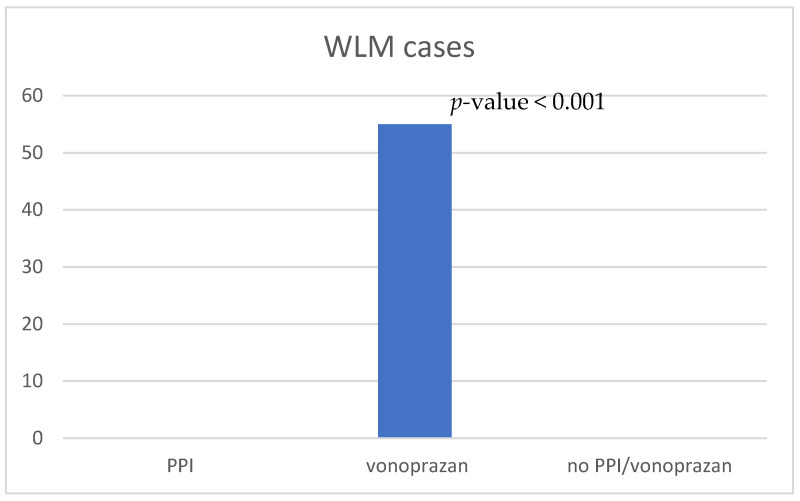
Comparison of the number of WLM occurrences. PPI, proton-pump inhibitor; WLM, web-like mucus.

WLM was observed in 55 patients in the vonoprazan-prescribed group (21%). None of the patients in the PPI- or no-PPI/vonoprazan-prescribed groups exhibited WLM (Figure 4), and there was a statistically significant difference compared to the vonoprazan-prescribed group (*p* < 0.001). Therefore, the patients in the vonoprazan-prescribed group were divided into two groups according to the presence or absence of WLM; their characteristics are shown in Table 1. There were no significant differences in sex, age, chronic kidney disease, diabetes mellitus, HP eradication history, smoking, alcohol consumption, or the use of antibiotics between the WLM and no-WLM groups. In both the WLM and no-WLM groups, antithrombotic drugs were used by 11 and 36 patients, respectively, and anticoagulants were used by 6 and 11 patients, respectively. The use of antithrombotic drugs tended to be slightly higher in the WLM group but without statistical significance.

**Figure 5:**
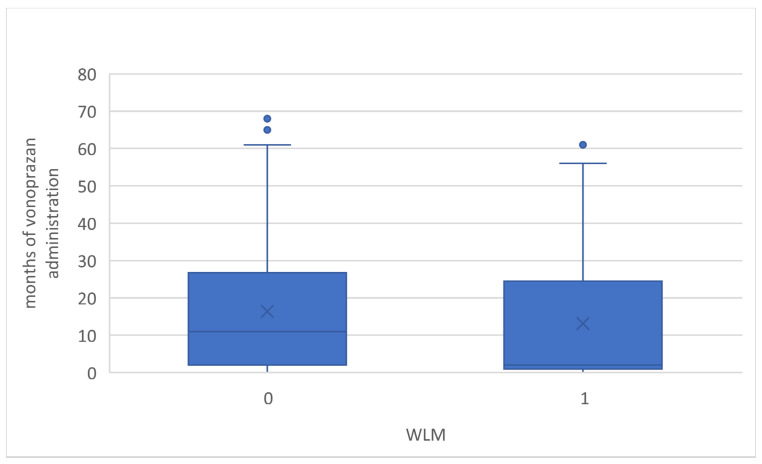
Box-plot of months of vonoprazan administration with and without WLM. WLM, web-like mucus.

The duration of vonoprazan administration could be retrospectively evaluated in 45 and 140 cases in both the WLM and no-WLM groups, respectively. The median duration from the start of vonoprazan administration to the endoscopy was 2 (1–24) and 11 (2–26) months, respectively, and there were no statistically significant differences (Figure 5). In eight cases in the WLM groups, vonoprazan was administered for less than a month, and, in the shortest case, it was only administered for 2 days. Furthermore, the multivariate logistic regression model was performed with adjustments for all potential confounding factors, as listed in Table 2.

### 3.2. Endoscopic Findings and Clinicopathological Evaluation

All WLM cases were observed in the upper gastric region, especially in the corpus; 16 and 11 cases were also observed in the middle and lower gastric regions, respectively. In contrast, no WLM was observed in the antrum. Fourteen and eighty-one patients in the WLM and no-WLM groups showed HP eradication, respectively (Table 1). Although the presence or absence of active HP infection could not be confirmed in all cases in this study, active HP infection was not suspected in any of the cases in the WLM group. The degree of endoscopic atrophic gastritis, which is closely associated with HP infection, did not differ between the WLM and no-WLM groups (Table 2).

Although the incidence of gastric mucosal redness and stardust gastric mucosa/white globe appearance lesions tended to be higher in the WLM group than in the no-WLM group (Table 2), there were no statistically significant differences.

## 4. Discussion

Vonoprazan exhibits a rapid, prolonged, and stronger inhibition of gastric acid secretion compared to PPIs [11]. Hence, vonoprazan is effective in various diseases and is a promising alternative to PPIs for the treatment of acid-related diseases [11]. Moreover, since its launch in Japan in 2015, the number of prescriptions has increased. As a result, the number of endoscopies in patients taking vonoprazan has increased. Since 2016, we have identified several patients with characteristic gastric mucus adhesions on endoscopy that distinctly differed from the purulent mucus found in HP infection and autoimmune gastritis cases. Furthermore, the mucus had white and transparent features, a spider web-like appearance, and was even more difficult to remove by washing with water injection than the sticky adherent dense mucus seen in autoimmune gastritis. Considering its appearance, we named this novel mucus “web-like mucus”. After repeatedly observing WLM, we noticed that all patients were taking vonoprazan. Thus, we considered that the WLM could be caused by vonoprazan, instead of PPIs or other causes. However, there have been no reports associating vonoprazan with increased gastric mucus.

**Figure 6:**
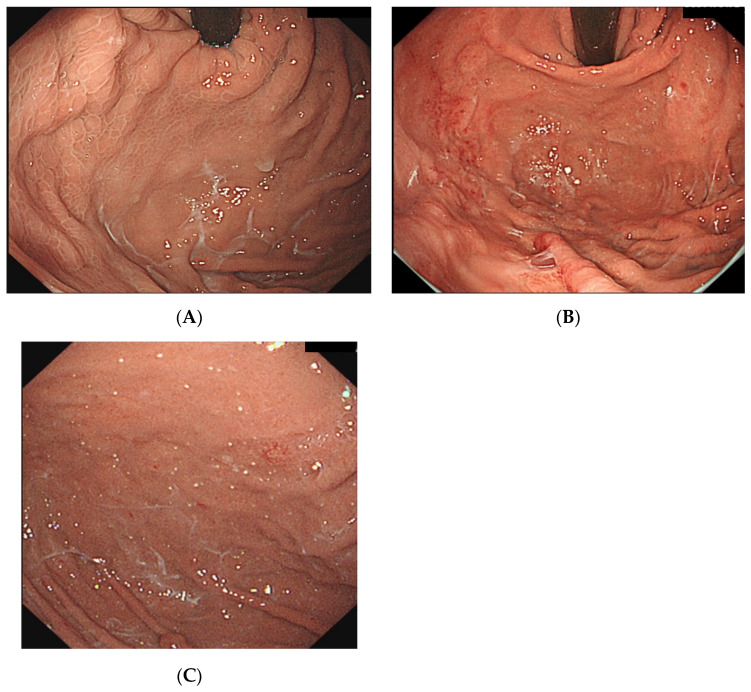
(**A**) WLM with cobblestone gastric mucosa. (**B**) WLM with gastric mucosal redness. (**C**) WLM with stardust gastric mucosa. WLM, web-like mucus.

However, recently, several studies have reported vonoprazan-associated endoscopic changes in the gastric mucosa, such as cracked and cobblestone-like gastric mucosa (Figure 6A), white globe appearance lesions in the noncancerous epithelium, stardust gastric mucosa (Figure 6B), and gastric mucosal redness (Figure 6C) [8,9,10,11]. Furthermore, the main exocrine cells in fundic glands are parietal cells, chief cells, and mucous neck cells, which produce gastric acid, pepsinogen, and mucus, respectively [14]. Thus, the inhibition of H^+^/K^+^-ATPase in the parietal cells of the fundic gland in a potassium-competitive manner by vonoprazan leads to the inhibition of gastric acid secretion [2,15,16]. In a previous study in dogs, vonoprazan inhibited gastric acid secretion by 8% after 24 h and 60% after 48 h [11]. Yoshizaki et al. suggested that due to the long-lasting and potent suppression of gastric acid secretion by vonoprazan, mucus secreted by mucous neck cells may be pooled in the fundic glands without being washed away by gastric acid and could be recognized as stardust gastric mucosa endoscopically [16]. In our study, although there was no difference in characteristic endoscopic findings with vonoprazan administration between patients with and without WLM, the incidence of stardust gastric mucosa/white globe appearance lesions tended to be higher in the WLM group than in the no-WLM group (Table 3). Considering Yoshikazi et al.’s suggestion, this excessive secretion of mucus could be a putative factor for WLM.

Mucus gel is generated by mucin granules through their apical expulsion from surface epithelial cells and contains 95% water and 5% mucin as products of mucin genes (MUC2, MUC5AC, MUC5B, and MUC6) [17]. The efficacy of the mucus depends on its gel structure and the thickness of the adherent mucus layer, and gastric mucin molecules are structurally bound to fatty acids, making them more hydrophobic [17,18]. The protective quality of gastric mucin is determined by the viscosity and permeability of the mucus layer enhanced by phospholipids [17,19]. Moreover, trefoil factor family (TFF) peptides, which are present in mucin secretory vesicles and are involved in intracellular assembly and packing of mucins [20], are expressed throughout the gastric mucosa. Thim et al. recognized that TFF2 increases the viscosity of the mucosal layer and stabilizes the gel network [21]. In our study, such relationships with mucus viscosity, mucus composition, and TFF peptides were not analyzed. However, the high viscosity of WLM makes it difficult to wash off with water injection. Thus, future studies should evaluate the detailed properties of WLM, as it is necessary to clarify the differences between WLM and other types of mucus, such as the sticky, adherent, dense mucus seen in autoimmune gastritis.

In contrast, examinations of gastric microbiota have been reported. Although gastric acidity plays a crucial role in filtering out bacteria and preventing the development of enteric infections, Del Piano et al. suggested that PPIs may affect the microbiota indirectly by reducing the acidity of the gastric environment, which in turn leads to gastric bacterial overgrowth [22]. Indeed, gastric acid suppression substantially increases the number of cultivable non-HP bacteria in both the gastric mucosa and the stomach lumen; notably, this effect is largely influenced by HP infection and the duration of acid suppression resulting from H2RA and PPIs [23]. Minalyan et al. also suggested that using PPIs favors relative streptococcal abundance irrespective of HP status, which may explain the persistence of dyspeptic symptoms in patients on PPI therapy [24]. As previously mentioned, vonoprazan exhibits a stronger inhibition of gastric acid secretion than PPIs [11]. Vonoprazan administration may also lead to an increase in cultivable non-HP bacteria in both the gastric mucosa and the stomach lumen. We performed biopsy cultures in WLM cases and confirmed the growth of bacteria such as *Streptococcus* species (Supplementary Materials). Although bacteria can travel down to the stomach from the oral cavity and *Streptococcus* species are also present as oral resident bacteria [25], whether these bacteria are part of a transient microbiome or permanent colonizers has been difficult to establish. Thus, the significance of bacterial overgrowth during acid-suppressive therapy depends on the ability of bacterial flora to contribute directly or indirectly to any pathologic condition. Although bacterial overgrowth is known to induce chronic inflammation and gastric malignancies and cancer can be induced by damage to the mucosal cells of infected individuals [22], there have been no detailed reports suggesting a relationship between mucus production and gastric bacteria. In this study, no common characteristic findings were found in biopsy specimens from the gastric mucosa with WLM, except for mild inflammation with lymphocytes, and there were no cases with gastric malignancies. Furthermore, this study did not analyze the microbiota of patients without WLM or those with PPI administration. Thus, the relationship between bacterial changes and WLM has not been elucidated. However, bacterial changes due to the crucial suppression of gastric acid secretion with vonoprazan administration may possibly be causally involved in WLM production and should be investigated in the future.

All WLM was found in the upper part of the stomach. In a previous study that used autoradioluminography to examine vonoprazan distribution in the rat stomach, vonoprazan was more localized in the gastric corpus than in the pylorus and antrum [26]. It was also reported that stardust gastric mucosa, a novel gastric finding potentially induced by vonoprazan, tended to be present in the upper third of the stomach [11]. Thus, although further investigation is required, the site of WLM attachment may also be associated with vonoprazan distribution.

In this study, no causal relationship between the duration of vonoprazan and the occurrence of WLM could be demonstrated statistically. Practically, there were patients in whom WLM appeared within a few days of the start of vonoprazan, while there were other patients in whom it took longer to appear. These results suggest that WLM may not occur in a dosing-period-dependent manner. No significant trends were observed in terms of age, sex, smoking, alcohol consumption, or use of antibiotics or antithrombotic drugs. Although the multivariate logistic regression model was performed with adjustments for potential confounding factors, such as sex, age, antibiotics, antithrombotic drugs, and duration of vonoprazan administration, there were no statistically causal relationships. Moreover, WLM may occur in non-HP-infected stomachs as well as in HP-eradicated stomachs. However, the vonoprazan-prescribed group included both cases with and without WLM. Therefore, further analysis is needed to elucidate the mechanisms causing WLM.

This study had some limitations. First, we did not evaluate all prescribed drugs other than PPIs, steroids, rebamipide, and NSAIDs. Although the percentage of use of antithrombotic drugs tended to be slightly higher in the WLM group, there were no statistically significant differences. In this study, because of more than 70% of patients with WLM did not use antithrombotic drugs, the causal relationship between the administration of antithrombotic drugs and WLM would appear to be negative. Second, we did not evaluate all foods taken within several days before the procedure. Third, this was a single-center, retrospective study performed by only two endoscopists. Despite these limitations, this study is the first to report a novel characteristic mucus observed in patients with vonoprazan administration. We hope that recognizing the characteristics of WLM will lead to its differentiation from other diseases that present with mucus adhesion, such as HP infection and autoimmune gastritis.

## 5. Conclusions

Gastric WLM may have occurred as a result of vonoprazan therapy. Furthermore, powerful suppression of acid secretion and bacterial growth might be involved in its generation. Thus, the fact that vonoprazan administration can produce a characteristic mucus is useful in differentiating it from other diseases, such as HP infection and autoimmune gastritis; however, the possibility of inadequate observation of the background gastric mucosa should be noted. The present study reports a preliminary observation of WLM, and we hope that further studies will be conducted to elucidate its pathogenesis, histology, and mechanisms.

## Figures and Tables

**Table 1:** Characteristics of the patients with and without WLM.

	WLM (*n* = 55)	Without WLM (*n* = 209)	*p*-Value
Sex (M/F)	28/27	112/97	0.763
Age, mean ± SD (years)	65.5 ± 11.5	65 ± 11.6	0.226
CKD (%)	3 (5%)	18 (9%)	0.582
DM (%)	9 (16%)	39 (18%)	0.845
HP eradication (%)	14 (25%)	81 (39%)	0.082
Smoking (%)	9 (16%)	34 (16%)	1.0
Alcohol consumption (%)	23 (42%)	80 (38%)	0.644
Antibiotics (%)	3 (5%)	4 (2%)	0.160
Rebamipid (%)	8 (14%)	43 (20%)	0.442
Steroids or other immunosuppressive agents (%)	6 (11%)	14 (7%)	0.388
NSAIDs (%)	3 (5%)	8 (4%)	0.703
Antithrombotic drugs (%)	15 (27%)	41 (20%)	0.196
Median no. of months of vonoprazan administration (IQR)	2 (1–24)	11 (2–26)	0.098

Abbreviations: WLM, web-like mucus; SD, standard deviation; M, male; F, female; CKD, chronic kidney disease; DM, diabetes mellitus; HP, *Helicobacter pylori*; NSAIDs, nonsteroidal anti-inflammatory drugs; IQR, interquartile range.

**Table 2:** Multivariate analysis of factors associated with WLM.

	OR	*p*-Value	95% CI
Male	0.724	0.399	0.342–1.534
Age	0.976	0.058	0.951–1.001
Antibiotics	4.161	0.273	0.325–53.193
Antithrombotic drugs	1.968	0.118	0.841–4.602
Duration of vonoprazan administration	0.990	0.418	0.968–1.014

Abbreviations: WLM, web-like mucus; OR, odds ratio; CI, confidence interval.

**Table 3:** Endoscopic characteristics of those with and without WLM.

	WLM (*n* = 55)	No-WLM (*n* = 209)	*p*-Value
Cobblestone gastric mucosa (%)	24 (44%)	92 (44%)	1.00
Gastric mucosal redness (%)	12 (22%)	36 (17%)	0.436
Atrophic gastritis (none/closed/open)	34/13/8	57/88/64	0.264
Multiple white and flat elevated lesions (%)	3 (5%)	13 (6%)	1.00
Stardust gastric mucosa/WGA (%)	8 (15%)	16 (8%)	0.120
Fundic gland polyps (%)	18 (33%)	44 (21%)	0.076
Hyperplastic polyps (%)	4 (7%)	24 (11%)	0.466

Abbreviations: WLM, web-like mucus; WGA, white globe appearance.

## Data Availability

The datasets are available from the corresponding author upon reasonable request.

## Supplementary Materials

The following supporting information can be downloaded at: https://www.mdpi.com/article/10.3390/jcm13144070/s1, Table S1. All bacteria detected in biopsy cultures of 16 cases with web-like mucus.
