# Supplementary material for: A Novel Characteristic Gastric Mucus Named “Web-like Mucus” Potentially Induced by Vonoprazan
Source: J Clin Med. 2024 Jul 11;13(14):4070. doi: 10.3390/jcm13144070 (PMC11277586; doi:10.3390/jcm13144070)
Supplement: Supplementary file 1 [file jcm-13-04070-s001.zip › jcm-2968678-supplementary.pdf]

Supporting information

Methods

In cases with web-like mucus (WLM), biopsy cultures were performed using the available mucus and mucosa samples to assess the presence or absence of bacteria.

Results

Bacterial flora in WLM

Gastric biopsies were performed on the mucous membranes of 16 patients, and bacterial cultures were obtained from 15 patients. *Streptococcus* species were the most common species, and *Escherichia coli* was the second most common (three cases). Supplementary Table 1 lists all the bacteria detected in this study. In addition, the histopathological findings showed mild inflammation with lymphocytes in the gastric mucosa in 16 cases, but no other specific findings were observed.

Table S1. All bacteria detected in biopsy cultures of 16 cases with web-like mucus.

	Streptococcus Genus	Others
1	*Streptococcus mitis, Streptococcus salivarius*	
2	*Streptococcus salivarius, Streptococcus vestibularis,* α-hemolytic *Streptococcus spp*	*Gemella haemolysans*
3	*Streptococcus mitis*	-
4	α-hemolytic *Streptococcus spp*	*Escherichia coli,* Aerobic Gram-positive *Bacillus*
5	*Streptococcus mitis*	-
6	*Streptocuccus parasanguinis*	*Escherichia coli*
7	*Streptococcus salivarius, Streptococcus mitis*	*Rothia mucilaginosa*
8	-	-
9	*Streptococcus mitis*	-
10	*Streptococcus mitis*	*Rothia mucilaginosa*
11	*Streptococcus mitis, Streptococcus salivarius*	-
12	*Streptococcus spp*	-
13	*Streptococcus mitis*	-
14	*Streptococcus oralis*	-
15	*Streptococcus mitis*	-
16	*Streptococcus salivarius*	*Escherichia coli*
